# Red Iron-Pigmented Tooth Enamel in a Multituberculate Mammal from the Late Cretaceous Transylvanian “Haţeg Island”

**DOI:** 10.1371/journal.pone.0132550

**Published:** 2015-07-15

**Authors:** Thierry Smith, Vlad Codrea

**Affiliations:** 1 Directorate Earth and History of Life, Royal Belgian Institute of Natural Sciences, Brussels, Belgium; 2 Faculty of Biology and Geology, University Babeş-Bolyai, Cluj-Napoca, Romania; Université de Poitiers, FRANCE

## Abstract

Mammals that inhabit islands are characterized by peculiar morphologies in comparison to their mainland relatives. Here we report the discovery of a partial skull associated with the lower jaws of a Late Cretaceous (≈70 Ma) multituberculate mammal from the Carpathian “Haţeg Island” of Transylvania, Romania. The mammal belongs to the Kogaionidae, one of the rare families that survived the Cretaceous—Paleogene mass extinction in Europe. The excellent preservation of this specimen allows for the first time description of the complete dentition of a kogaionid and demonstration that the enigmatic *Barbatodon transylvanicus* presents a mosaic of primitive and derived characters, and that it is phylogenetically basal among the Cimolodonta. Another peculiarity is the presence of red pigmentation in its tooth enamel. The red coloration is present on the anterior side of the incisors and on the cusps of most of the teeth. Energy-dispersive X-ray spectrometry (EDS) analysis reveals that the pigmented enamel contains iron, as in living placentals. Such a red pigmentation is known in living soricine shrews and many families of rodents, where it is thought to increase the resistance of the enamel to the abrasion that occurs during “grinding” mastication. The extended pattern of red pigment distribution in *Barbatodon* is more similar to that in eulipotyplan insectivores than to that in rodents and suggests a very hard diet and, importantly, demonstrates that its grasping incisors were not ever-growing. As inferred for other endemic Transylvanian vertebrates such as dwarf herbivorous dinosaurs and unusual theropod dinosaurs, insularity was probably the main factor of survival of such a primitive mammalian lineage relative to other mainland contemporaries of the Northern hemisphere.

## Introduction

Mammals that inhabit islands are characterized by peculiar morphologies in comparison to their mainland relatives [1]. This is also the case in the fossil record, with examples including the Pleistocene dwarf Flores man from Indonesia [2], the 90 cm high elephant *Palaeoloxodon falconeri* from Sicily [3], the pigmy mammoth *Mammuthus exilis* from California’s Channel Islands [4], the Pliocene-Holocene extremely hypsodont (high-crowned teeth) endemic bovid *Myotragus* from the Balearic Islands [5], and the Miocene gigantic hedgehog *Deinogalerix* and strange small five-horned ruminant *Hoplitomeryx* from the Gargano Peninsula in Italy [6,7].

Deeper in time, during the Late Cretaceous, Southern Europe was an archipelago [8]. High sea levels and warm temperature were responsible of the fragmentation of this European paleobioprovince into numerous small islands [9,10]. On one of them, the so-called “Haţeg Island” [11], the Late Cretaceous Transylvanian vertebrates from Romania formed an island paleofauna [12–14]. Geological and paleobiogeographical data indicate that “Haţeg Island” in fact corresponded to an area more extended than the Haţeg Basin itself, and included also the Transylvanian Basin, the Rusca Montană Basin, and surrounding areas [12,15–18]. The “Haţeg Island” has been estimated to cover an area of approximately 80,000 km^2^, corresponding to the size of the Caribbean island Hispaniola [12]. In this particular environment, island-dwelling mammals cohabited with endemic dwarf and unusual dinosaurs [19–21]. Because no other mammal groups than kogaionid multituberculates have been identified, with the possible exception of one tooth of an undetermined therian [22], the Haţeg fauna has been interpreted as an evolutionary cradle for the enigmatic kogaionids that spread across Western Europe during the Paleocene [13]. However, due to the paucity of well-preserved specimens, particular features of these insular mammals have never been characterized.

**Fig 1:**
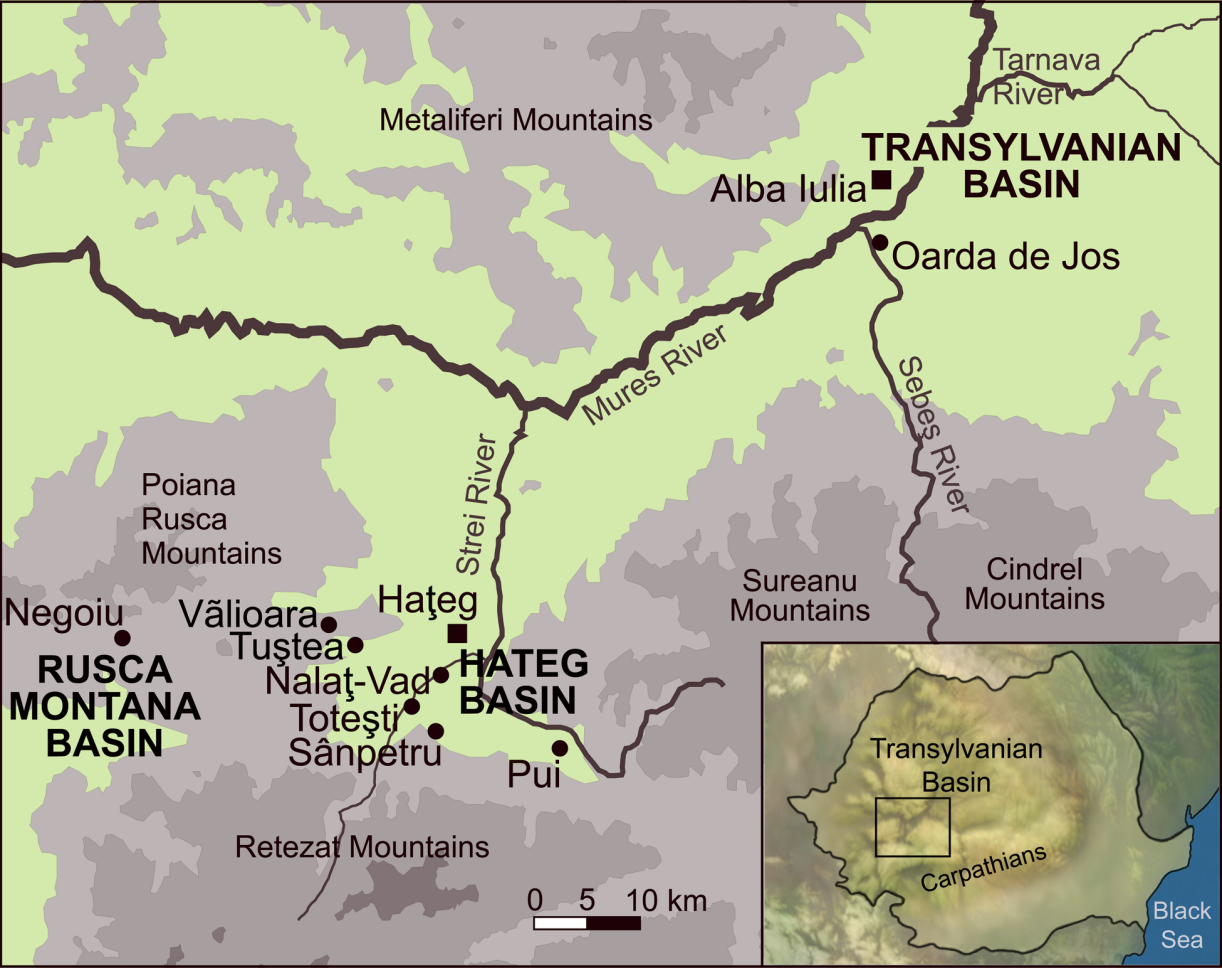
Location of the principal Maastrichtian continental localities of the Transylvanian Basin, Hateg Basin, and Rusca Montana Basin (Romania) that have yielded kogaionid multituberculate mammal remains (black dots). The specimen UBB P-Mt1 of *Barbatodon transylvanicus* and the holotype tooth are both from the locality of Pui.

We report here new dental and cranial remains of an island-dwelling kogaionid multituberculate mammal of about 70 million years old, discovered in the Maastrichtian deposits of Transylvania (Fig 1). These specimens, which present red tooth enamel, predate the oldest previously known red-toothed mammals by about 15 million years, extending the record of such teeth into the Mesozoic. The teeth and bones of the specimens are white except for the blood-red areas of the dentition that are demonstrated here to contain iron concentration, as in some living insectivores and rodents, revealing an especially hard diet.

## Materials and Methods

### Material collected

Fossil specimens described here (UBB P-Mt 1, 4–1, 4–2 and 4–3) are stored at the University Babeş-Bolyai of Cluj-Napoca, Laboratory of Paleotheriology and Quaternary Geology, Romania. All necessary permits were obtained for the described study, which complied with all relevant regulations. The specimens were discovered at the locality of Pui (Fig 1, S1 Fig). They were extracted from the silty matrix and prepared at the vertebrate laboratory of University Babeş-Bolyai. Final preparation was done at the microvertebrate laboratory of the Royal Belgian Institute of Natural Sciences.

### Cladistic analysis

A cladistics analysis was performed using the heuristic algorithm of TNT 1.1 [23], based on the most utilized matrix for multituberculates [24]. A more recent matrix [25], based on Kielan-Jaworowska and Hurum’s matrix to which were added about 40% characters retrieved from other studies, was tested but not used here because several characters were erroneously coded. Another very recent study has partially improved Yuan et al.’s matrix [26] but there are still character codes that do not correspond to the morphology described in the literature. As an example, characters 19 and 20 of Yuan et al.’s matrix (corresponding respectively to characters 3 and 4 in Kielan-Jaworowska and Hurum’s matrix), which concern the number of cusps on I2 and I3 were erroneously coded for over 50% of the taxa. Fearing further coding mistakes, we kept the classical Kielan-Jaworowska and Hurum’s matrix supplemented with *Barbatodon* reported here and *Hainina*. Character definitions of *Kogaionon* are identical to theirs except character 1 (enamel microstructure) which was originally coded as? and here replaced by state 1 (gigantoprismatic) because this character state was identified later [27], and character 39 (molar enamel ornamentation) which was coded as 0 and here replaced by state 1 because M2 presents grooves and ridges. All characters were considered unordered.

### SEM-EDS analysis

Due to the high scientific value and the rarity of a nearly complete specimen of a European Cretaceous mammal, enamel composition was analyzed by a non-destructive technique. Pigmented enamel composition was identified on surface with a low environmental scanning electronic microscope (ESEM Quanta 200) in conjunction with EDS (EDAX Apollo 10). For comparison with other mammals bearing pigmented teeth, we also sampled extant soricine *Sorex araneus* and murid *Rattus norvegicus* following the same methodology.

## Results

### Systematic Paleontology

Class Mammalia Linnaeus, 1758

Subclass Allotheria Marsh, 1880

Order Multituberculata Cope, 1884

Suborder Cimolodonta McKenna, 1975

Family Kogaionidae Rădulescu & Samson, 1996

Genus *Barbatodon* Rădulescu & Samson, 1986

*Barbatodon transylvanicus* Rădulescu & Samson, 1986

(Fig 2)

**Fig 2:**
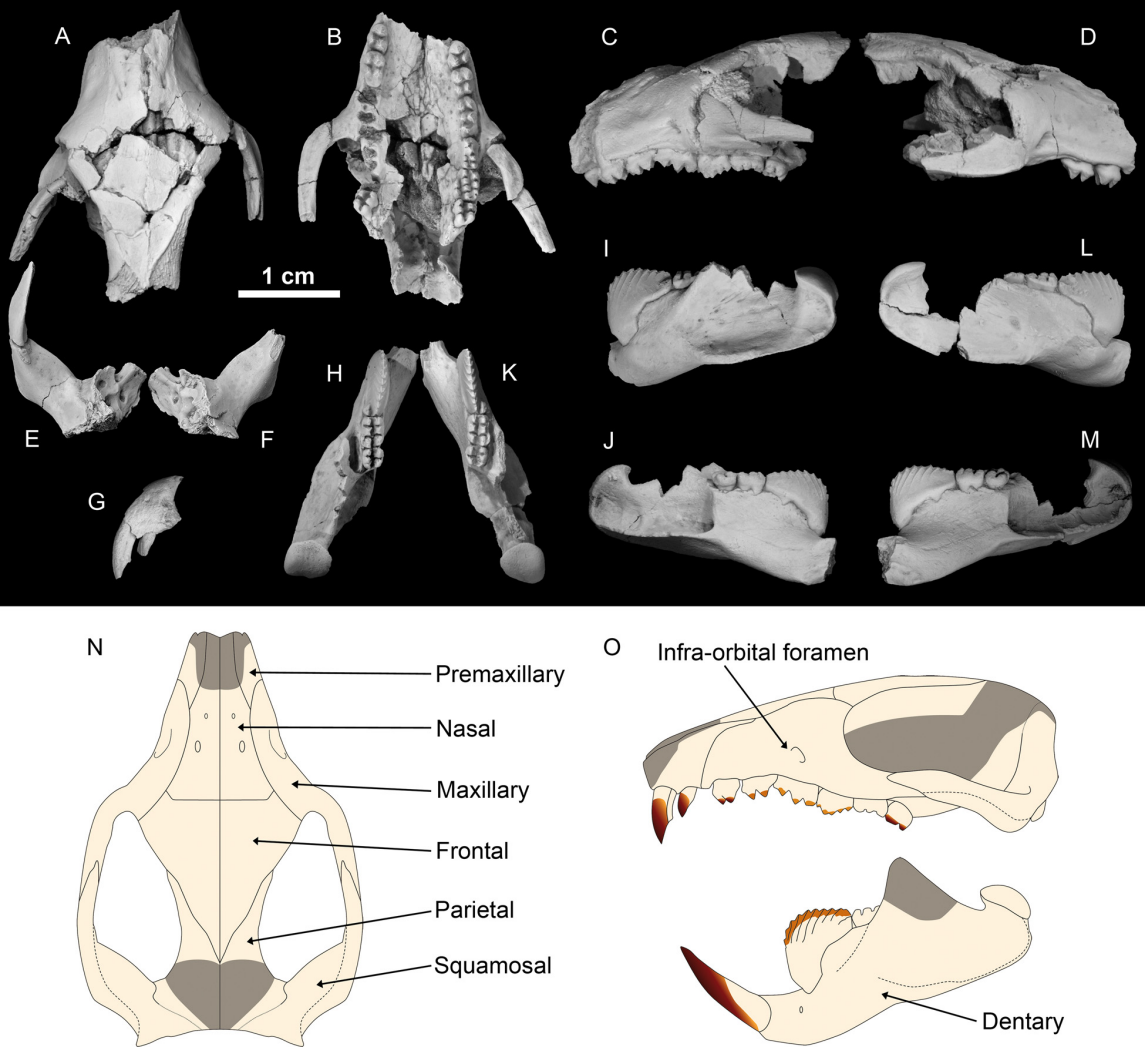
*Barbatodon transylvanicus*, Maastrichtian, Pui, Romania, specimen UBB P-Mt 1. Partial skull in (A) dorsal, (B) ventral, (C) left lateral, (D) right lateral views; Left squamosal and petrosal (E) and right petrosal (F) in dorsal view; Left premaxillary in (G) lateral view; Left dentary in (H) occlusal, (I) labial, (J) lingual views; Right dentary in (K) occlusal, (L) labial, (M) lingual views. Specimens covered with ammonium chloride. Reconstruction of the skull and dentary in (N) dorsal and (O) lateral views. Grey zones indicate missing parts.

#### Holotype

ISB IS.001 (Institute of Speleology “Emil Racoviţă”, Bucharest, Romania), an isolated left m1.

#### Referred specimens

UBB P-Mt 1, a rostrum with anterior part of zygomatic arches, the left premaxillary (with I2-3), the left squamosal, both petrosals, and both associated dentaries (with p4-m2). UBB P-Mt 4–1, I2; UBB P-Mt 4–2, I3; UBB P-Mt 4–3, partial i1.

#### Type Locality and Age

Pui locality, along the Bărbat River in the Bărbat Valley of the eastern part of the Haţeg Basin, southern Carpathian Mountains of southwestern Transylvania, Romania (Fig 1, S1 Fig). The deposits belong to the Sânpetru Formation, which represents the Early-Late Maastrichtian (Late Cretaceous) transition, based on palynostratigraphy and magnetostratigraphy (S2 Fig) [28–30].

#### Description and comparison

The best fossil specimen here described is a partial skull of the kogaionid cimolodontan *Barbatodon transylvanicus* represented by the rostrum that preserves the anterior part of the zygomatic arches, the left premaxilla, the left squamosal, both petrosals, and both associated dentaries (specimen UBB P-Mt 1, Fig 2). It shows the diagnostic characters of the genus *Barbatodon* [31,32] and especially those of the species *Barbatodon transylvanicus*, which was originally described on the basis of the specimen ISB IS.001, an isolated left m1 from the same locality of Pui [33,34]. Knowledge of the lower dentition of this species was enhanced with the discovery of specimen FGGUB M.1635 (Faculty of Geology and Geophysics, University of Bucharest, Bucharest, Romania), two dentaries associated with an isolated right M2, also from the Pui locality [31]. The dentition of the new specimen described here exhibits exactly the same characters, such as the large arcuate p4 with 9–10 cusps, the short and wide m1 with a cusp formula 3–4:3, and the square shaped M2; it is thus referred to *Barbatodon transylvanicus*. A few differences exist between UBB P-Mt 1 and the specimens of *B*. *transylvanicus* previously described that we consider here as intraspecific variability. The length of m1 is 10% shorter than in ISB IS.001 and FGGUB M.1635 but the width is the same. The posterior part of the dentary between the coronoid process and the condyle is a little shallower than in FGGUB M.1635 and the p4 is somewhat longer.

Allocation of *Barbatodon transylvanicus* to the Multituberculata and more precisely to the suborder Cimolodonta is confirmed by the presence of only two upper incisors, four upper premolars, an arcuate p4, and the absence of p1-3 [24]. This species belongs to the family Kogaionidae, as indicated by the elongated upper premolars, especially P3, a short and wide M1 with only four cusps in the middle row, and a p4 that protrudes dorsally over the level of the molars (measurements: see Table 1).

**Table 1:** Measurements of the teeth (in mm) of the kogaionid multituberculate *Barbatodon transylvanicus* from Pui (specimen UBB P-Mt 1).

*Barbatodon transylvanicus*				
Specimen number	Description	Position	Measurements	
			length	width
UBB P-Mt 1	left maxilla	P1	2.60	2.10
		P2	3.20	2.10
		P3	4.20	2.10
		P4	3.30	1.90
		M1	3.20	2.50
		M2	2.30	2.30
		P1-4	12.20	
		M1-2	5.50	
		P1-M2	17.20	
	right maxilla	P1	2.70	2.10
		P2	3.20	2.10
		M2	2.40	2.30
	left dentary with P_4_-M_2_	P_4_	7.60	2.20
		M_1_	3.00	2.20
		M_2_	2.00	2.10
		M_1-2_	5.00	
		P_4_-M_2_	11.90	
	rigth dentary with P_4_-M_2_	P_4_	7.60	2.20
		M_1_	3.10	2.20
		M_2_	2.00	2.10
		M_1-2_	5.10	
		P_4_-M_2_	12.00	

The upper dentition of *B*. *transylvanicus* is here identified for the first time, thus allowing a comparison with the dentition of other kogaionids and especially that of *Kogaionon ungureanui*, the only other European Late Cretaceous mammal that is known based on a partial skull [35]. *Barbatodon transylvanicus* differs from *Kogaionon ungureanui* (Sânpetru locality, Tămăşel Hill, Sibişel Valley, central part of the Haţeg Basin, Sânpetru Formation) by its smaller size (15% smaller), longer frontal bones, absence of diastema between I2 and I3, more rectangular M1 with only two cusps in the lingual row instead of three, more square M2 instead of triangular, P4 that has about the same width throughout the length of the tooth and bears two cusps on the lingual row (P4 of *K*. *ungureanui* is wider on the posterior border and presents a shorter lingual row), and P2 that lacks a long posterior expansion. *B*. *transylvanicus* differs from *B*. *oardaensis* (Oarda de Jos locality, Alba County, southwestern part of the Transylvanian Basin, Şard Formation) by its much larger size (50% larger) and by having two cusps in the labial row of P3 instead of three [32]. *B*. *transylvanicus* differs from all species of *Hainina* (*H*. *belgica*, *H*. *godfriauxi*, *H*. *pyrenaica*, *H*. *vianeyae*), all from the Paleocene, by its larger size and a simpler morphology with a lower number of cusps on P3-M1.

A detailed description of the craniodental morphology of *Barbatodon* will be provided in a separate study as this paper mainly focuses on its phylogenetic position and its peculiar tooth pigmentation.

### Phylogenetic analysis

**Fig 3:**
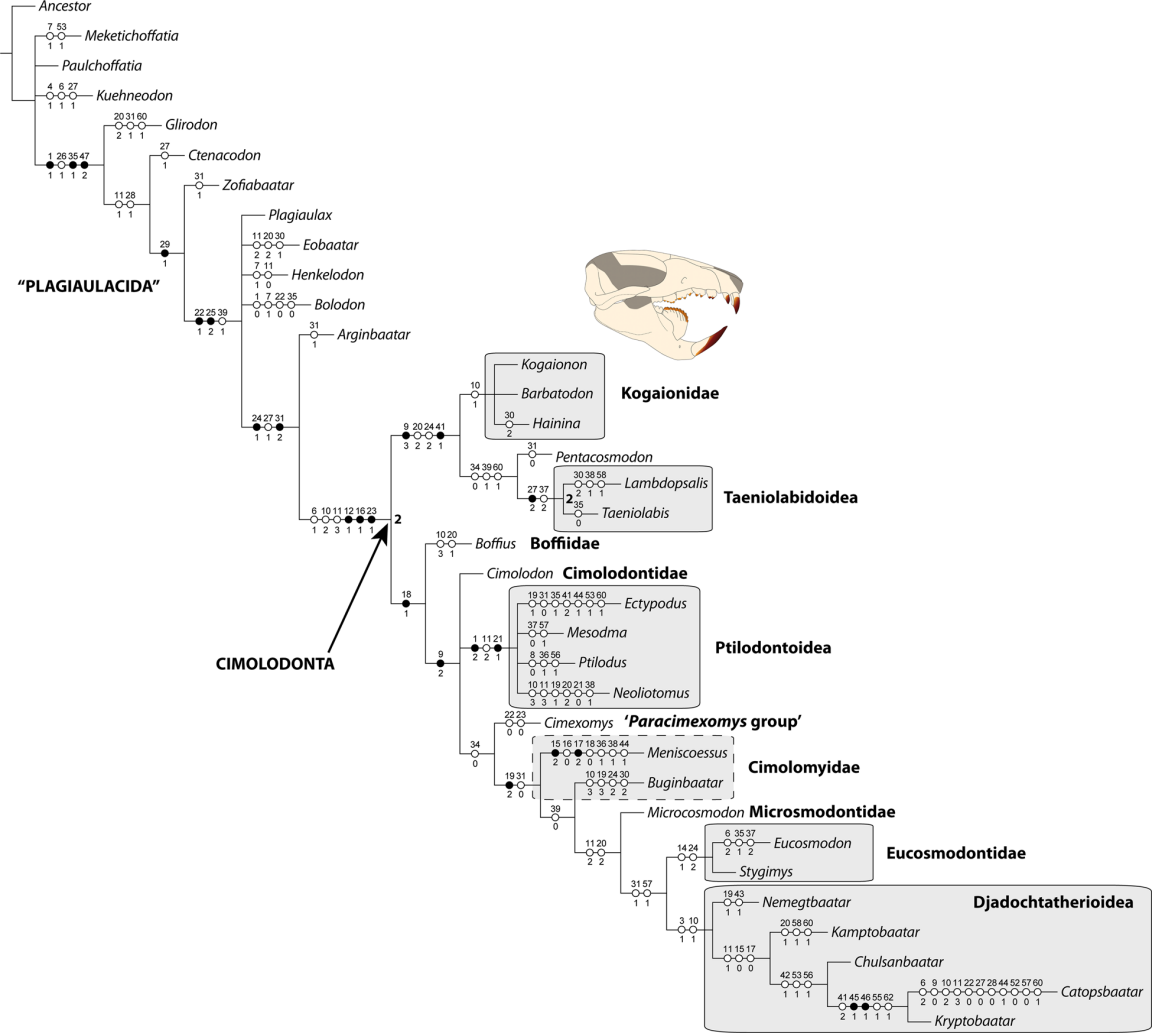
Strict consensus cladogram of the 355 equally most parsimonious trees showing the position of the Kogaionidae. The topology is obtained from the cladistic analysis of the matrix from Kielan-Jaworowska & Hurum 2001 with addition of *Barbatodon* and character 1 of *Kogaionon* which was originally coded? and here replaced by state 1. Tree length, 215 steps; CI, 0.417; RI, 0.663. For each node, the list of the synapomorphies is given, each synapomorphy being represented by a point (black for unambiguous synapomorphy and white for homoplasies) accompanied by the character number above and character state below. Bremer decay indices of 2 and higher are indicated at the right of their respective nodes.

The character coding of *Barbatodon transylvanicus*, based on the skull from Pui (specimen UBB PMt 1) and dentaries (specimens UBB P-Mt 1 and FGGUB M.1635), is as follow:? 11011003131001100120112—11-12001100000122000200????1?101?010. The character coding of *Hainina*, based on data from the literature [36–39] is as follow: 1????1003131?0???01???????11?22001?0001???????????????????????. The equally-weighted parsimony analysis of 62 dental and cranial characters (52 of which could be scored for *Barbatodon* and 22 for *Hainina*) based on the most utilized matrix for multituberculates [24] yielded 355 equally parsimonious trees of 215 steps (CI: 0.442; RI: 0.696). The topology of the strict consensus (Fig 3) is relatively similar to that obtained by Kielan-Jaworowska and Hurum [24] but better resolved. All families of Cimolodonta, except Cimolomyidae, are monophyletic. *Barbatodon* and *Hainina* are grouped together with *Kogaionon* and, unlike in previous works [24,25,26,40], Kogaionidae are grouped with Taeniolabidoidea and *Pentacosmodon*, and placed at the root of the clade Cimolodonta. *Kogaionon* is also one of the most basal cimolodontan multituberculates in the recent independent analysis of Yuan et al. [25]. Nevertheless, it is in a terminal position in a first analysis of Mao et al. but again in a basal position in a second analysis with 19 characters being ordered [26].

### Tooth pigmentation analysis

**Fig 4:**
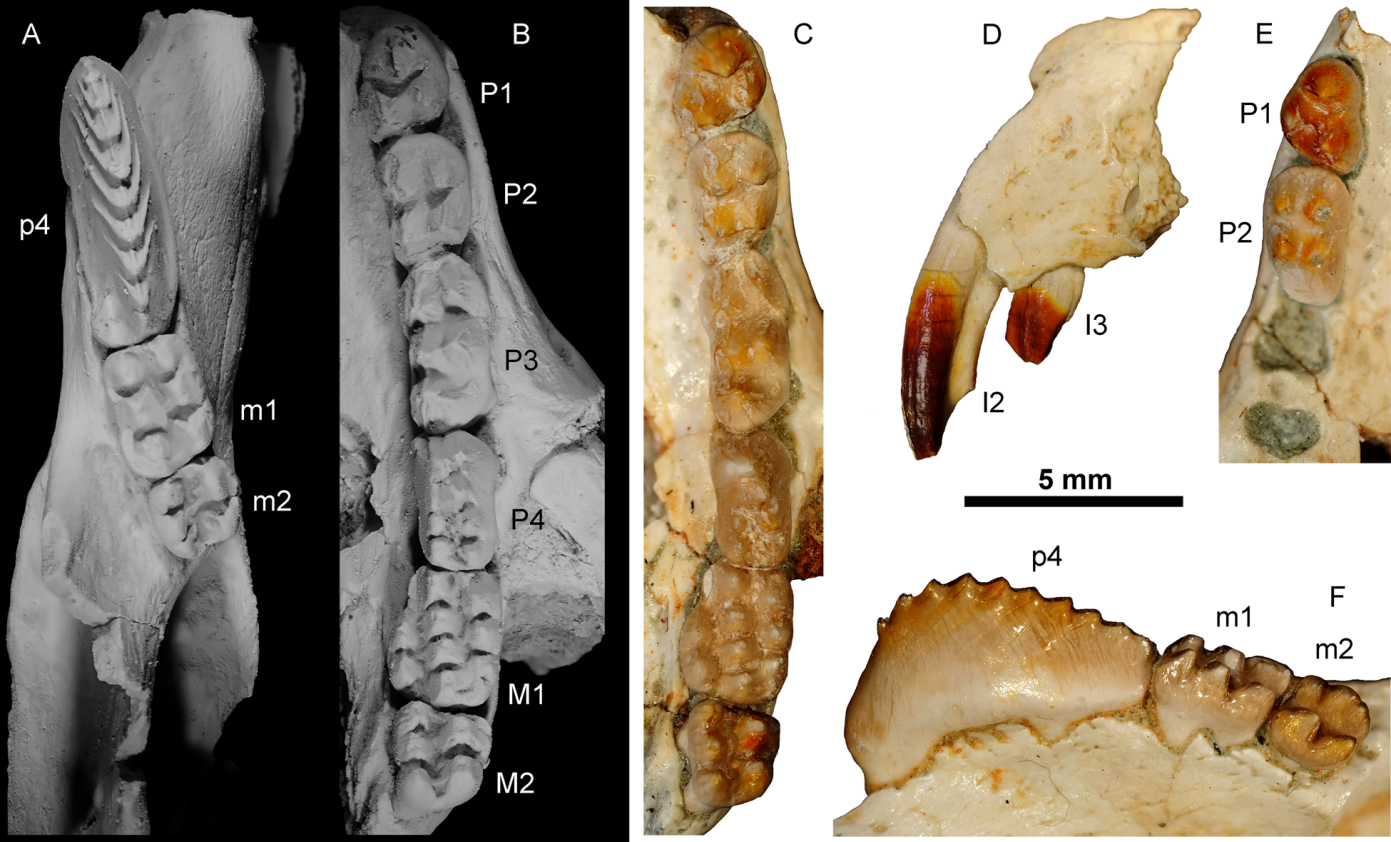
Close-up on tooth rows of *Barbatodon transylvanicus*. (A) Left dentary with p4-m2 and (B) left maxillary with P1-M2 in occlusal views and covered with ammonium chloride. (C) left maxillary with P1-M2 in occlusal view; (D) Premaxillary with in I2-3 in lateral view; (E) anterior part of right maxillary with P1-2 and (F) right dentary with p4-m2 in lingual view in natural colour.

**Fig 5:**
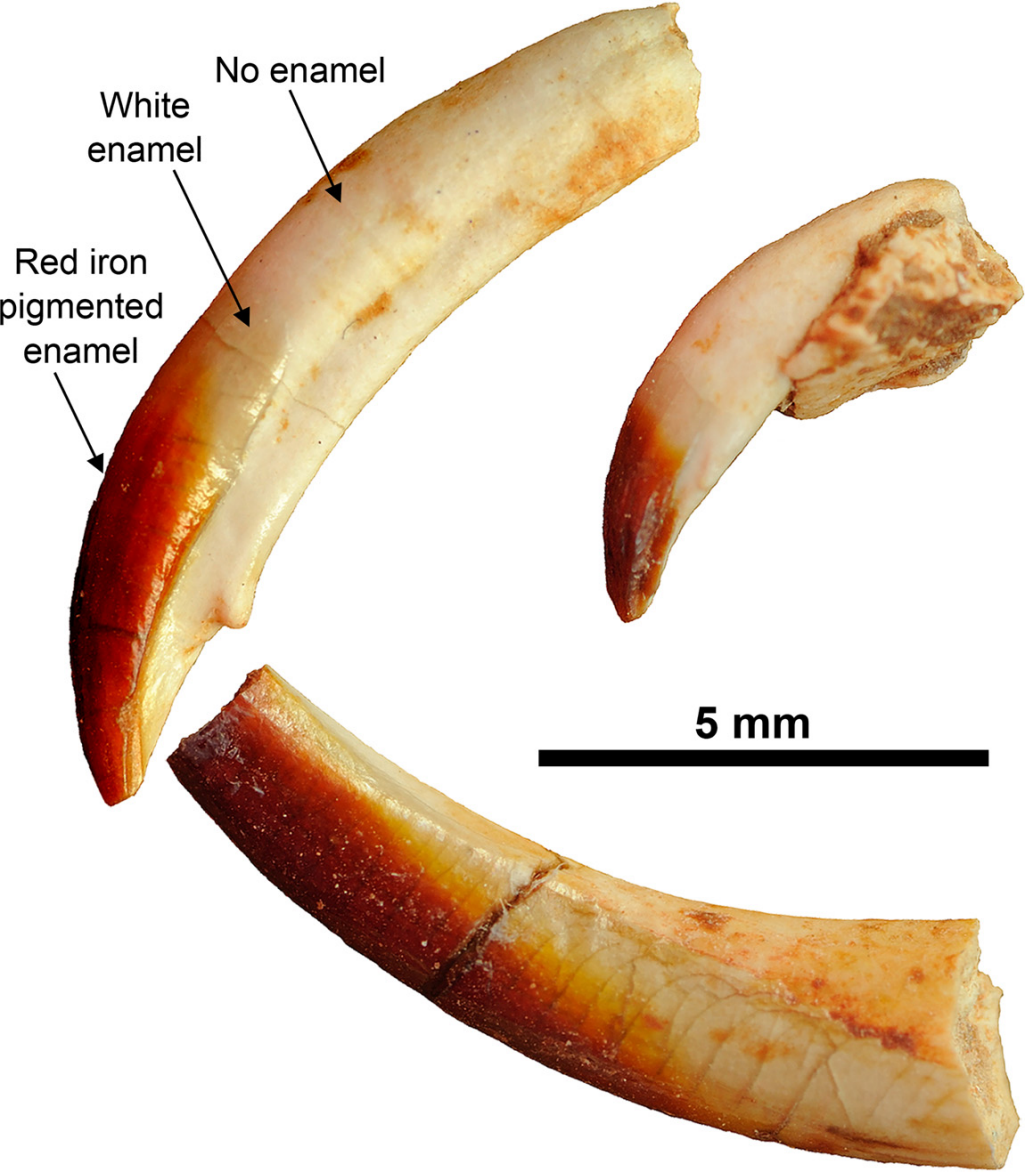
Isolated incisors of *Barbatodon transylvanicus* from Pui. I2 (UBB P-Mt 4–1), I3 (UBB P-Mt 4–2), and partial i1 (UBB P-Mt 4–3) show that the enamel is not extended on all the length and that only a part of the enamel is red.

All of the tooth positions of *B*. *transylvanicus* (specimens UBB P-Mt 1, 4–1, 4–2 and 4–3) have reddish to red cusps and some teeth are more reddish than others (Figs 4 and 5). The reddest teeth are the two upper incisors (I2 and I3). P1 and M2 are also more reddish than P2 to M1. On the lower teeth, the upper edge of p4 and the m2 are more reddish than m1. The most reddish teeth are thus the front teeth and the back teeth. The teeth situated toward the middle of the dental row (P4-M1; posterior part of p4 and m1) are weakly reddish.

**Fig 6:**
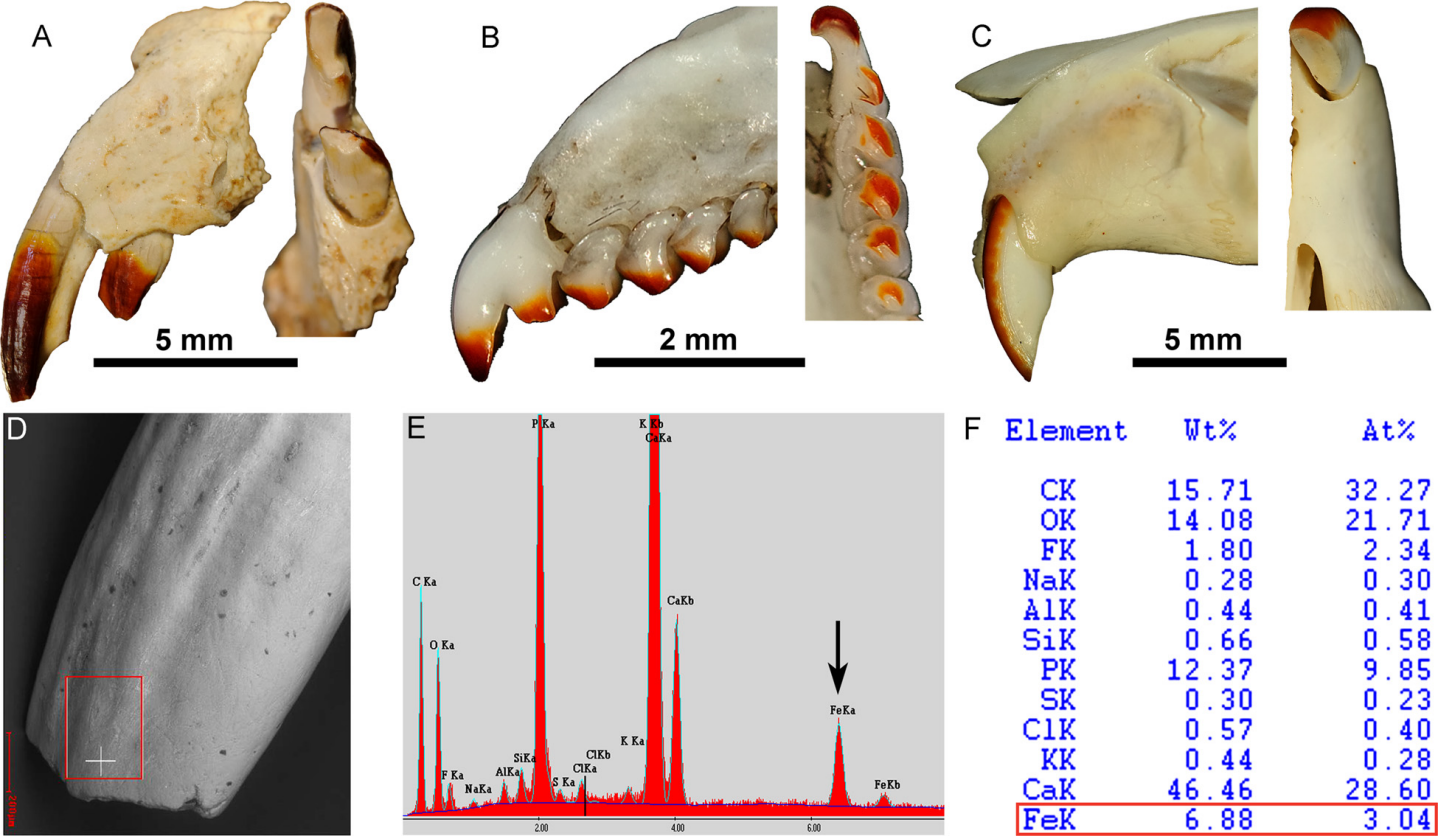
SEM-EDS analysis of red enamel. Close-up on the left premaxillary of (A) *Barbatodon transylvanicus*, (B) *Sorex araneus* and (C) *Rattus norvegicus* in lateral and occlusal views showing the red enamel on the anterior surface of the teeth. Analysis of the enamel of *Barbatodon transylvanicus* on the (D) anterior tip of I1 in (E) EDS analysis showing the (F) elemental composition of Calcium hydroxyapatite and a peak of about 7% (3% in atomic composition) of iron.

Comparison of iron concentration by EDS indicates that the most reddish enamel areas incorporated more iron. The most reddish tooth is the large upper incisor (I2), which has a blood red colour containing 6.88% (3.04% in atomic composition) of iron at the tip (Fig 6). Incisors of the extant Soricine *Sorex araneus* and murid *Rattus norvegicus* also contain more iron than other teeth with a maximum for the tested specimens of 7.78% (3.06%) and 5.61% (2.25%) respectively (S3 and S4 Figs).

## Discussion

Specimen UBB P-Mt 1 of *Barbatodon transylvanicus* represents the most complete and best-preserved of any Late Cretaceous mammal of Europe. Based on the nominally informative holotype, the genus *Barbatodon* was tentatively assigned to the North American *Paracimexomys* group [24,34,41]. The present study, based on a more complete specimen, confirms assignment of *Barbatodon* to the family Kogaionidae, which belongs to the basalmost clade of cimolodontan multituberculates (Fig 3). One of the peculiarities of kogaionids is thus the presence of particularly primitive characters relative to other mainland contemporaries of the Northern hemisphere (e.g., North American taeniolabidoids and ptilodontoids and Asian djadochtatherioids). However, none of the phylogenetic analyses of multituberculates is robust and the addition of few character coding from a single new specimen can change de configuration of the phylogenetic tree.

Our study resolves a long controversy about the validity of *Kogaionon ungureanui* with respect to *B*. *transylvanicus*, the first being known on the basis of a partial skull with upper teeth and the second mainly known from the lower teeth [31,41]. Despite their closely similar morphologies, both names are thus valid and represent two distinct kogaionid taxa. This should also allow identification in the near future of new kogaionid taxa on the basis of tens of isolated teeth of previously unidentified species from several Transylvanian localities [18,38,42–44]. Interestingly, kogaionid mammals survived the Cretaceous-Paleogene (K-Pg) mass extinction, as represented by species of *Hainina*, which are known from typical isolated teeth from the early Paleocene of Spain and Belgium [36,39], and the late Paleocene of France and Romania [38,45]. The only other mammal group that is known to have also survived the K-Pg crisis in Europe is the arboreal eutherian family Adapisoriculidae [46,47].

*Barbatodon transylvanicus* is also the first Mesozoic mammal known to have teeth with red enamel. It is noteworthy to recall that, among shrews, the subfamilies Soricinae and Myosoricinae have red teeth whereas Crocidurinae do not [48]. These shrews display strong coloration of their enamel on the tips of tooth cusps of all dental positions, including the incisors. Some red-toothed shrews are even venomous (e.g., the North American *Blarina brevicauda*, the Eurasian *Neomys fodiens*, and the Mediterranean *Neomys anomalus*). Like the insectivorous *Solenodon* from the Caribbean islands, they are capable of injecting toxic saliva but only the latter developed a narrow channel located on the lingual surface of the second lower incisor that functions like a hypodermic needle to deliver venom [49]. A similar venom delivery apparatus has also been suggested in the Paleocene pantolestid *Bisonalveus browni* [50]. Rodents also have reddish pigmentation, but only on the enamel band of the labial side of the incisors. Among living rodents, all of the major groups (i.e., Hystricomorpha, Castorimorpha, Sciuromorpha, and Myodonta) have members with red incisors [51].

Although these red-pigmented teeth have been nominally studied in living mammals, their elemental composition has never been identified in fossil mammals. In most cases, coloration of teeth and bones changes during the fossilization process depending on the composition of the sediment that contains the fossil remains. However, some rare fossil taxa have preserved original tooth pigmentation. The oldest red teeth documented in soricids are found in *Domnina gradata* from the early Oligocene of North America [48]. Pigmentation on cusp tips similar to that of soricines is known in the very high-crowned teeth of the Eocene zalambdodont insectivore *Apternodus* [52]. Until now, the oldest records of red tooth preservation date back to the late Paleocene Asian taeniolabidoid multituberculates *Lambdopsalis bulla* and *Sphenopsalis nobilis*, which have pigmented enamel on the ventrolateral surface of the incisors and the crown of the second upper and lower molars [26,53,54].

Our results indicate that the blood-red coloration of teeth of *B*. *transylvanicus* is due to concentration of iron oxides in the enamel that thus represents the oldest record of iron-pigmented teeth in mammalian evolution. In both insectivores and rodents, the pigmented layer is composed of iron directly contained in the matrix of the superficial aprismatic enamel [55,56]. The red enamel in mammals consists of discrete layers of iron oxides deposited external to unpigmented hydroxyapatite enamel [57] and is thought to increase the resistance of the enamel to mechanical stress and to acid dissolution [58,59]. Teeth and parts of teeth that are subject to greatest stresses, excessive wear and to fracture present a higher density of iron [60]. This relationship between the quantity of iron incorporated in teeth and the hardness of the diet has also been studied in groups other than mammals, such as in the radular teeth of the limpet *Patella* (Gastropoda) and the chiton *Acanthopleura* (Polyplacophora), and the teeth of the axolotl *Ambistoma* (Amphibia) and several fishes such as piranhas (Characidae) and butterflyfishes (Chaetodonidae) [61–65]. Among the latter, a quantitative analysis has revealed significant differences among eight butterflyfish species that appear to be related to their feeding ecology: the specialized hard-coral browser *Chaetodon ornatissimus* has a higher iron concentration in its most resistant teeth [64].

Two main functions are recognized for iron pigmented teeth in modern mammalian taxa. One is to prolong the life of the tooth in soricines. Quantitative study on *Blarina brevicauda* demonstrated that the hypoconid cusp incorporates a higher concentration of iron than any other cusps [60]. The hypoconid includes the primary crushing and grinding surface and therefore is more prone to excessive wear than the shearing surfaces. The second is the primary development of cutting edges, as is the case of rodent incisors. Rodents have ever-growing incisors and the pigmentation serves to harden the enamel and maximize the differential wear between the enamel and the dentine, which produces a sharp, chisel-like tooth [60]. Recently, nano-analytical techniques combined with high resolution techniques such as STEM, synchrotron X-ray diffraction and X-ray photoelectron spectroscopy have determined that the red enamel pigmentation of *B*. *brevicauda* is due to ultrafine magnetite Fe_3_O_4_ grains deposited around the hydroxyapatite crystals, which suggests that iron is probably released by ferritin granules during a pigmentation release stage of amelogenesis [66]. Moreover, the authors demonstrated by nanoindentation measurements that the pigmented enamel is on average 30% harder than unpigmented enamel [66]. Another recent study on rodents has shown the presence of poorly crystalline ferrihydrite in the pigmented enamel of the North American beaver *Castor canadensis* [67]. This suggests that pigmented enamel in shrews and rodents results from a convergent evolution as it does not involve the same iron oxide.

The function of an iron-rich enamel in the Cretaceous multituberculate *Barbatodon* is more difficult to elucidate. Despite the general rodent-like aspect of the skull and incisors, the comparison of the extended pattern of distribution of enamel pigmentation in *Barbatodon* with that in Eulipotyphla and rodents indicates more similarities to soricine shrews than to rodents. The red coloration of the cusps of the front premolars and the back teeth suggests that they were associated with crushing and grinding, which are typical features of a very hard diet. A hard diet is also suggested by the strong wear of some teeth of *Barbatodon transylvanicus* [31] as well as in other kogaionid species from different European faunas [39,44]. More importantly, as in *Sorex*, red pigmentation is not distributed all along the incisor of *Barbatodon* like it is in *Rattus* (Fig 4). The enamel is only present on the anterior part of the tooth and the iron pigmentation is absent from the basal part of the incisors indicating that the incisors were not ever-growing (Fig 5). Indeed, the red enamel band of ever-growing incisors of rodents extends across the whole crown of the tooth, including the part enclosed in the alveolous within the jaw. However, it is relevant to note that the end of the root of I2 UBB P-Mt 4–1 of *Barbatodon* is not strongly tapered and closed off while it is on I3 UBB P-Mt 4–2 (Fig 5). As such, it could be that the central incisors were indeed not ever-growing but that they grew until late in life before closing off. The inclusion of iron in the enamel would have slowed the wear on the tooth.

These results have to be highlighted with the fact that multituberculates underwent an adaptive radiation starting at least 20 million years prior to the K-Pg boundary. The disparity in their dental complexity was related to a range of diets, and this dietary expansion apparently tracked the ecological rise of angiosperms [68]. The escalation in angiosperm vein density from mid-Cretaceous to earliest Cenozoic, visible in angiosperm fossil leaves but not in non-angiosperm seed plants and ferns [69], may have affected the diet of cimolodontan multituberculates, even though small taxa probably were not folivores. Although their dentition was specialized for herbivory, they probably were not strictly herbivorous [25,70,71]. Some families of multituberculates are remarkable for the development of a blade-shaped p4 that is interpreted to be used during an orthal slicing-crushing cycle for processing of food objects [71]. The kogaionid *Barbatodon* has an especially well developed blade-shaped p4 and small lower molars like the North American ptilodontid *Ptilodus* that has been suggested as omnivorous [72]. Curiously, the taeniolabidoid multituberculate *Lambdopsalis bulla* that also has red teeth [53] and gigantoprismatic enamel [73] has very different tooth morphology with very small p4 and large molars. This difference suggests that red pigmentation presents at the tips of the large p4 of *Barbatodon*, which is much less concentrated than on m2/M2, was probably not directly related to the breaking of resistant food objects but just for a better resistance of the enamel like for the other tooth positions.

In conclusion, *Barbatodon transylvanicus* was an insular primitive multituberculate with a very hard diet and red iron pigmented teeth showing a pattern distribution similar to eulipotyphlan insectivores and grasping incisors that were not ever-growing. Like for other vertebrate groups, “Hateg Island” would have served as a refugium for the kogaionid lineage, which then evolved in isolation until at least the K-Pg boundary and therefore retained an unusual number of primitive features. The insularity was thus probably the main factor of survival of such a primitive mammal lineage, similarly to the islander survivor from an archaic terrestrial mammalian lineage that inhabited New Zealand archipelago between the Late Cretaceous and early Miocene [74]. The gondwanatherian *Vintana* showing a combination of primitive and highly derived features recently described from the Maastrichtian of Madagascar [75] is another example of insular mosaicism in an allotherian mammal but from Southern Hemisphere.

## Supporting Information

S1 Fig Location (red arrow) of the discovery of the specimen UBB P-Mt1 of the kogaionid multituberculate *Barbatodon transylvanicus* along the Barbat River in the Barbat Valley at Pui. (TIF)

S2 Fig Age constraint of the fossiliferous deposits of the Sânpetru Formation to the Early-Late Maastrichtian transition. Based on palynology [44] and magnetostratigraphy [45–46]. Modified from [44].
(TIF)

S3 Fig Energy-dispersive X-ray analysis in conjunction with low environmental scanning electronic microscope of the upper incisor of *Sorex araneus*. A pike of iron is visible on the reddish part of the enamel. The red square on the SEM picture indicates the precise location of the analysis on the dark red tip of the incisor.
(TIF)

S4 Fig Energy-dispersive X-ray analysis in conjunction with low environmental scanning electronic microscope of the upper incisor of *Rattus norvegicus*. A pike of iron is visible on the anterior reddish band of the enamel. The red square on the SEM picture indicates the precise location of the analysis near the tip of the incisor.
(TIF)
