# Supplementary figures and images for: Red Iron-Pigmented Tooth Enamel in a Multituberculate Mammal from the Late Cretaceous Transylvanian “Haţeg Island”
Source: PLoS One. 2015 Jul 15;10(7):e0132550. doi: 10.1371/journal.pone.0132550 (PMC4503309; doi:10.1371/journal.pone.0132550)

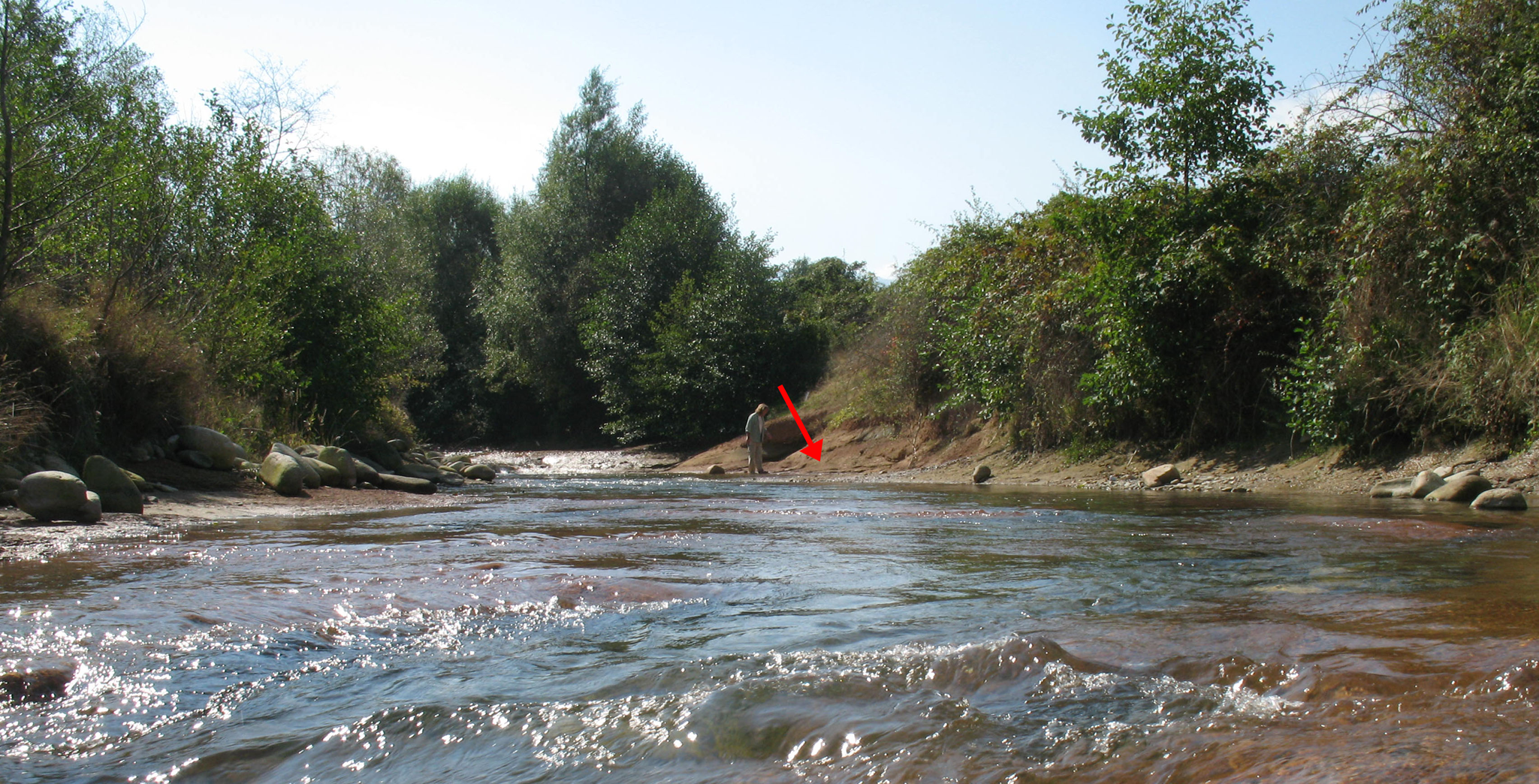

Supplement: S1 Fig — (TIF) [file pone.0132550.s001.tif]

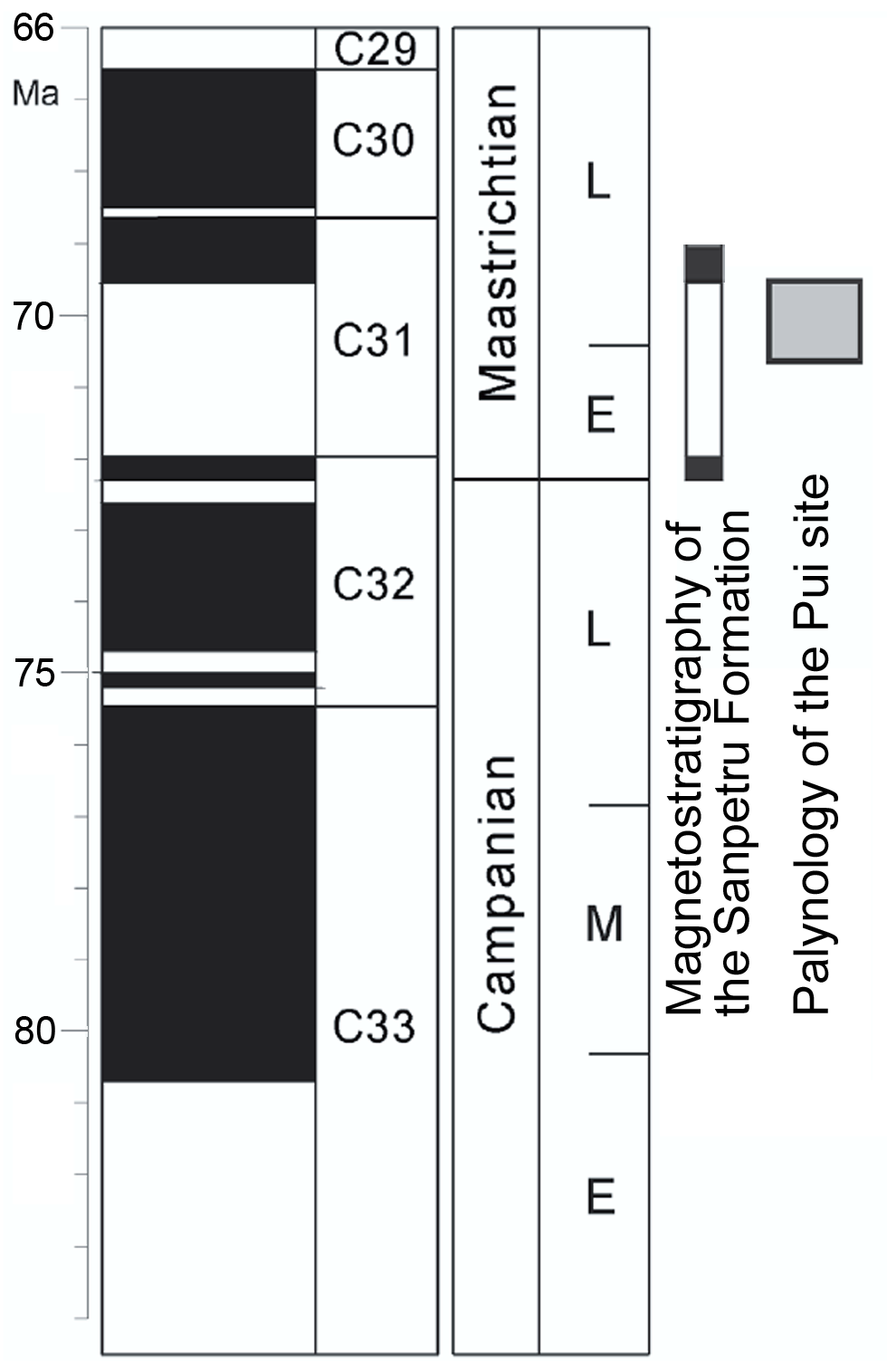

Supplement: S2 Fig — Based on palynology [44] and magnetostratigraphy [45–46]. Modified from [44]. (TIF) [file pone.0132550.s002.tif]

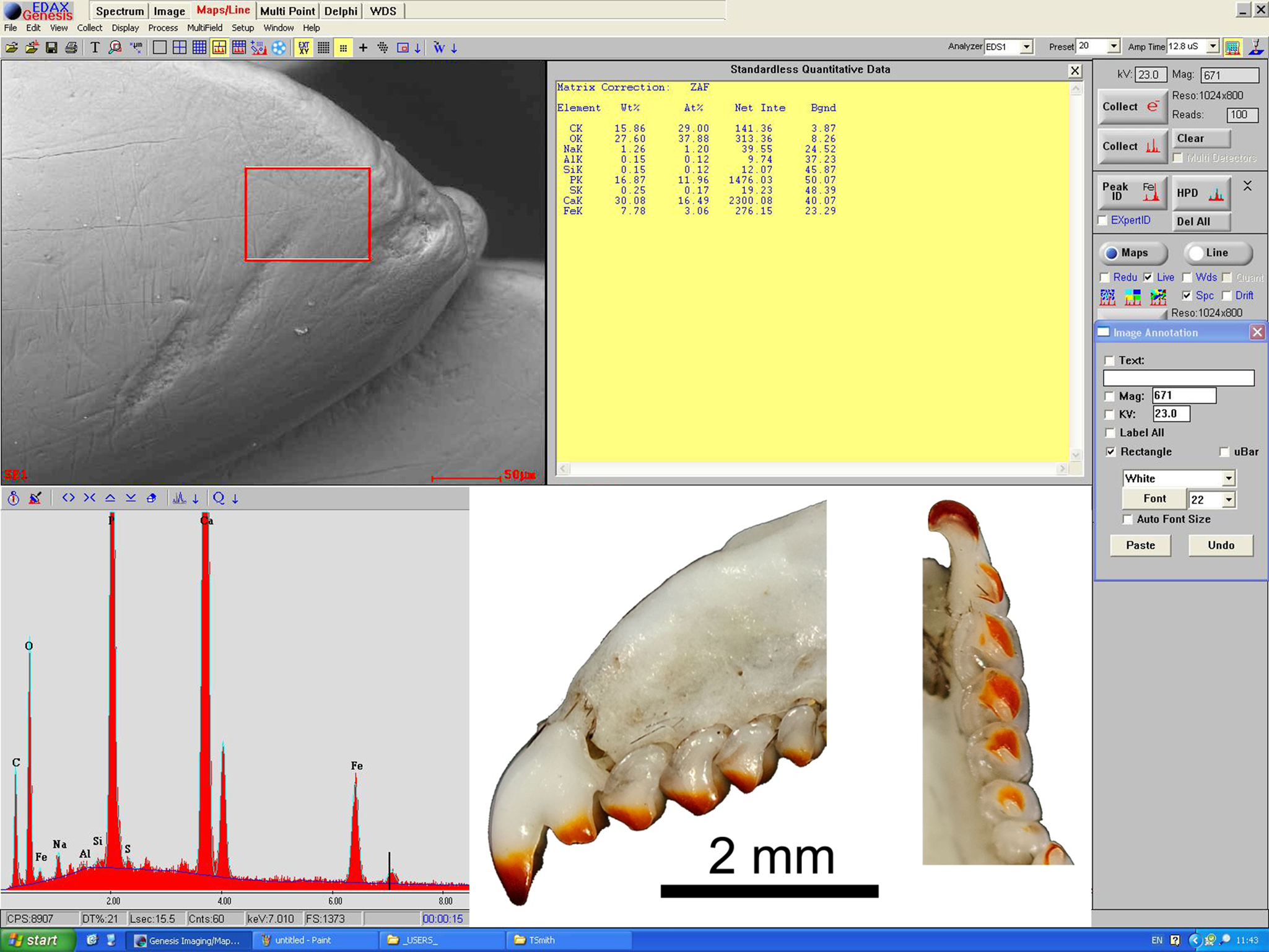

Supplement: S3 Fig — A pike of iron is visible on the reddish part of the enamel. The red square on the SEM picture indicates the precise location of the analysis on the dark red tip of the incisor. (TIF) [file pone.0132550.s003.tif]

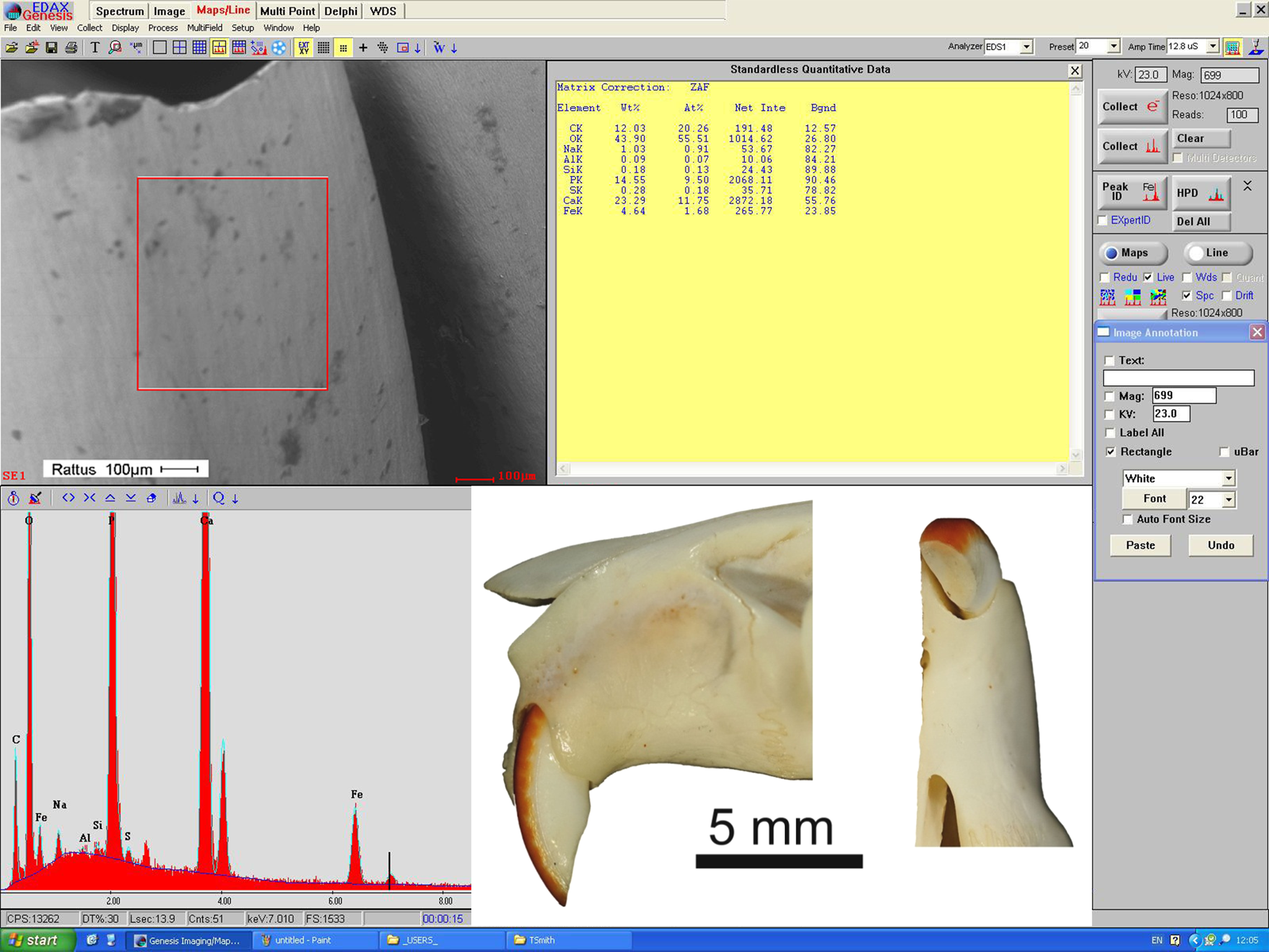

Supplement: S4 Fig — A pike of iron is visible on the anterior reddish band of the enamel. The red square on the SEM picture indicates the precise location of the analysis near the tip of the incisor. (TIF) [file pone.0132550.s004.tif]
